# Hypertension control in integrated HIV and chronic disease clinics in Uganda in the SEARCH study

**DOI:** 10.1186/s12889-019-6838-6

**Published:** 2019-05-06

**Authors:** Dalsone Kwarisiima, Mucunguzi Atukunda, Asiphas Owaraganise, Gabriel Chamie, Tamara Clark, Jane Kabami, Vivek Jain, Dathan Byonanebye, Florence Mwangwa, Laura B. Balzer, Edwin Charlebois, Moses R. Kamya, Maya Petersen, Diane V. Havlir, Lillian B. Brown

**Affiliations:** 1 Infectious Diseases Research Collaboration, Kampala, Uganda; 2 University of California San Francisco, San Francisco, CA USA; 3 University of Massachusetts, Amherst, MA USA; 4 Makerere University College of Health Sciences, Kampala, Uganda; 5 University of California, Berkeley, CA USA

**Keywords:** Hypertension, HIV/AIDS, Sub-Saharan Africa, Integrated care

## Abstract

**Background:**

There is an increasing burden of hypertension (HTN) across sub-Saharan Africa where HIV prevalence is the highest in the world, but current care models are inadequate to address the dual epidemics. HIV treatment infrastructure could be leveraged for the care of other chronic diseases, including HTN. However, little data exist on the effectiveness of integrated HIV and chronic disease care delivery systems on blood pressure control over time.

**Methods:**

Population screening for HIV and HTN, among other diseases, was conducted in ten communities in rural Uganda as part of the SEARCH study (NCT01864603). Individuals with either HIV, HTN, or both were referred to an integrated chronic disease clinic. Based on Uganda treatment guidelines, follow-up visits were scheduled every 4 weeks when blood pressure was uncontrolled, and either every 3 months, or in the case of drug stock-outs more frequently, when blood pressure was controlled. We describe demographic and clinical variables among all patients and used multilevel mixed-effects logistic regression to evaluate predictors of HTN control.

**Results:**

Following population screening (2013–2014) of 34,704 adults age ≥ 18 years, 4554 individuals with HTN alone or both HIV and HTN were referred to an integrated chronic disease clinic. Within 1 year 2038 participants with HTN linked to care and contributed 15,653 follow-up visits over 3 years. HTN was controlled at 15% of baseline visits and at 46% (95% CI: 44–48%) of post-baseline follow-up visits. Scheduled visit interval more frequent than clinical indication among patients with controlled HTN was associated with lower HTN control at the subsequent visit (aOR = 0.89; 95% CI 0.79–0.99). Hypertension control at follow-up visits was higher among HIV-infected patients than uninfected patients to have controlled blood pressure at follow-up visits (48% vs 46%; aOR 1.28; 95% CI 0.95–1.71).

**Conclusions:**

Improved HTN control was achieved in an integrated HIV and chronic care model. Similar to HIV care, visit frequency determined by drug supply chain rather than clinical indication is associated with worse HTN control.

**Trial registration:**

The SEARCH Trial was prospectively registered with ClinicalTrials.gov: NCT01864603.

**Electronic supplementary material:**

The online version of this article (10.1186/s12889-019-6838-6) contains supplementary material, which is available to authorized users.

## Background

There is an increasing non-communicable disease (NCD) burden globally, with an estimated one billion people living with hypertension and about 9.4 million related deaths annually [1]. Global trends are mirrored in sub-Saharan Africa (SSA) [2–4] where hypertension has become a major public health problem [5]. Population surveys of prevalence reveal a large burden of undiagnosed and untreated hypertension across SSA [6–8], and suggest that even among individuals in care, hypertension is successfully controlled in less than a quarter [7].

HIV is more prevalent in SSA than anywhere else in the world [9] and the region is facing a dual HIV-NCD epidemic. The expansion of life-saving antiretroviral therapy (ART) has decreased HIV related morbidity and mortality [10, 11], leading to an aging population living with HIV who are more susceptible to NCDs such as hypertension [12, 13]. As concern about the management of NCDs among people living with HIV (PLHIV) grows, the infrastructure that has been built for the provision of ART and other care services must be leveraged and adapted to respond to the growing burden of NCDs among both PLHIV and HIV-uninfected populations.

In this setting, lessons learned from the HIV chronic disease treatment model can be applied to the management of other chronic diseases such as hypertension. Integrated care systems are more convenient for patients, decrease stigma associated with healthcare, and could be more efficient for government and non-governmental funders. However, evidence-based care models for scaling up integrated HIV/NCD care are lacking [14]. In particular, little is known about the health systems factors that might influence HTN control when leveraging HIV chronic care systems to provide care for persons with hypertension, with or without co-occurring HIV infection. In the HIV chronic care model, systems factors such as clinic waiting times, inconvenient clinic hours, and unfriendly attitudes from staff, and frequency of schedule visits are associated with patient engagement in care and clinical outcomes [15]. In addition, drug stock outs for cardiovascular medications are frequent in SSA [16] and visit frequency, which is often determined by drug supply, is associated with clinical outcomes in the HIV chronic care model [17, 18].

In the Sustainable East Africa Research in Community Health (SEARCH) study (NCT01864603), population level screening of HIV and HTN was performed at community health campaigns, and individuals with either or both diseases were linked to integrated care at local health facilities [19, 20]. The integrated care model addressed many of the known structural barriers to engagement in care through flexible clinic hours with decreased wait times, patient-centered care, and welcoming attitudes. In this study we set out to: 1) characterize the patient population and HTN control over time among of adult residents who linked to HTN care using an integrated chronic care delivery model that offered treatment for both HTN and HIV disease and 2) evaluate predictors of HTN control over time.

## Methods

### Study setting

We studied 10 rural Ugandan communities participating in the intervention arm of the SEARCH Study. Communities selected for the SEARCH study in Uganda met initial eligibility criteria of a rural community, defined as one or more national geopolitical units, just above the village level (i.e. a parish) with a population of about 10,000 persons within the catchment area of a President’s Emergency Plan for AIDS Relief (PEPFAR)-supported HIV clinic in southwestern Uganda or Eastern Uganda and matched pairs were selected based on region, population density, occupational mix, access to transport routes, and number of trading centres [21]. Among the approximately 10,000 persons residing in each community, approximately 50% are adults age ≥ 18 years. Following a baseline census, each community held a community health campaign (CHC) offering multi-disease screening, treatment and linkage to care. Point-of-care screening for HIV, hypertension, and diabetes was offered to all adults (age ≥ 18 years) [19] and persons screening positive for any condition were linked to care at a nearby health center. All HIV-positive persons were offered the first line regimen in Uganda at the time of efavirenz, tenofovir disoproxil fumurate, and emtricitabine [22].

### Hypertension definition

We defined HTN based on World Health Organization (WHO) guidelines as a systolic BP ≥ 140 or diastolic BP ≥ 90 mmHg on any one of three measurements [23] or self-reported current use of anti-hypertensives. Stage 1 hypertension was defined as highest systolic BP > = 140 mmHg and < 160 mmHg OR highest diastolic BP ≥ 90 mmHg and < 100 mmHg. Stage 2 hypertension was defined as highest systolic BP ≥ 160 mmHG or DBP ≥ 100 mmHg. Hypertension control was defined as systolic BP < 140 AND diastolic BP < 90 mmHg on all three blood pressure measurements.

### Clinic procedures

Participants who screened positive for hypertension at the CHC were referred to their local health facility for NCD management. An integrated chronic disease model of streamlined care designed to reduce patient level barriers and maximize health system efficiency [20] was implemented at all local clinics. HIV and NCD care were co-located and HIV care was part of a chronic disease care model that offered joint evaluation and management of hypertension, diabetes, and general medical conditions. HIV-infected patients received HIV and NCD-focused care simultaneously during their visit. HIV-uninfected persons received treatment for hypertension and/or diabetes.

At the clinic visit blood pressure was measured using electronic sphygmomanometers. Individuals with hypertension were managed using a clinical and medication management algorithm based on the Uganda Clinical Guidelines [24] [Additional files 1 and 2]. Patients with Stage 1 hypertension were initially managed with a 3-month trial of lifestyle changes. If a patient’s blood pressure remained elevated after that trial, then the patient was prescribed blood pressure lowering medication and scheduled to return in 4 weeks. On subsequent visits, individuals with uncontrolled blood pressure were scheduled to return to clinic 4 weeks later for repeat blood pressure check and medication titration if necessary. Per algorithm, those with controlled blood pressure were scheduled to follow-up in 3 months. However, in practice, patients with controlled blood pressure were often scheduled to return to clinic earlier (e.g. after 4–6 weeks rather than the 3 months indicated) due to drug stock outs. All patients with malignant hypertension (BP > 180/110 mmHg) were referred immediately to the clinical officer for urgent treatment at the health facility.

### Outcome

The primary outcome of interest for this analysis was hypertension control at follow-up clinic visits. Blood pressure control at 2 consecutive visits separated by at least 1 month was evaluated as a secondary outcome.

### Analysis

We described univariate distributions of demographic and clinical variables overall and separately among HIV-infected and uninfected individuals. Demographic characteristics of those who linked to care were compared with those who screened positive for HTN at CHC and did not link to care using logistic regression adjusting for clinic site. We classified a scheduled visit as ‘more frequent than clinical indication’ if blood pressure was controlled and the next clinic visit was scheduled within 84 days. Late visits were defined as visits made more than 14 days after the scheduled date.

We used a multilevel mixed-effects logistic regression [25] to evaluate our hypothesis that scheduled visits more frequent than clinical indication would be associated with worse blood pressure control at follow-up visits. We identified covariates a priori for inclusion in multivariate analysis based on known factor related to blood pressure control. In final multivariate analysis we adjusted for individual characteristics (age, sex, comorbid diabetes based on chart review, HIV-status at baseline), clinic, time-varying clinical characteristics (hypertension stage at previous visit, medications prescribed at previous visit), and calendar time (follow-up time in months). Clinic site was modeled as a fixed effect because we were interested in quantifying between-clinic heterogeneity and to control for the depends of all individuals within a clinic. Individuals were modeled as random effects to account for intra-individual correlation in the outcome. All individuals who had at least one follow-up visit were included in the analysis. All analyses were conducted with Stata version 14.2 (Statacorp LP, College Station, Texas).

### Ethics

This study and all consent procedures were approved by ethical review boards of Makerere University School of Medicine (Kampala, Uganda) and the University of California, San Francisco (USA). All participants provided verbal informed consent at the CHC in their preferred language with fingerprint biometric confirmation of agreement. Verbal consent was provided in lieu of written consent as the CHC activities presented no more than minimal risk of harm to subjects and did not involve procedures for which written consent would otherwise be provided, and because of limited literacy in the study population.

## Results

### Population screening and linkage to care

**Fig. 1:**
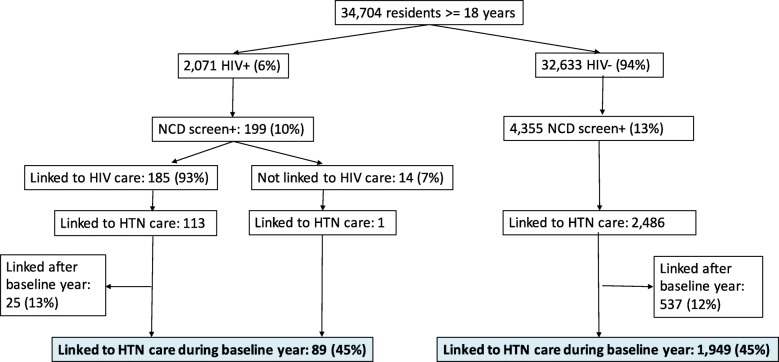
Population screened for HIV and hypertension and linked to hypertension care within one year in the SEARCH Study

Across ten communities, 34,704 residents aged ≥18 years were evaluated at baseline, of whom 2071 (6%) were HIV-infected [Fig. 1, Table 1]. Among HIV-infected adults, 199/2071 (10%) screened positive for hypertension. Among HIV-uninfected individuals 4355/32,633 (13%) screened positive.

**Table 1:** Demographic characteristics of individuals who linked to hypertension care after population-based screening at their baseline clinic visitn (*N* = 2038)

	HIV+ (*n* = 89)	HIV- (*n* = 1949)	Overall (*N* = 2038)
Sex			
Male [n(%)]	27 (30%)	820 (42%)	847 (42%)
Female [n(%)]	60 (70%)	1129 (58%)	1189 (58%)
Age [Median (IQR)]	46 (40, 54)	56 (45, 69)	56 (45, 68)
BMI [Median (IQR)]	22.2 (20.4, 25.0)	22.4 (20.0, 25.9)	22.4 (20.0, 25.8)^a^
On antihypertensive medication [n(%)]	10 (11%)	284 (15%)	294 (15%)
Stage 1 Hypertension [n(%)]	32 (36%)	746 (38%)	778 (38%)
Stage 2 Hypertension [n(%)]	32 (36%)	682 (35%)	714 (35%)
Diabetes	4 (4%)	119 (6%)	123 (6%)
HIV RNA < = 500 copies/mL [n(%)]	31 (34.8%)	NA	NA

^a^ missing data in *n* = 89 (4%)

Within one year, 45% (2038/4554) of individuals who screened positive for hypertension linked to NCD care during the baseline year [Fig. 1]. Among those who linked, 69% (1189/2038) were women, with a median age of 56 years (IQR 45–68 years) and median BMI of 22.4 (IQR 20.0–25.8). Participants who screened positive for hypertension and linked to care were more likely to be female (52% vs 43%, *p* < 0.001), older (mean age 55.5 vs 48.8 years, *p* < 0.001), and have Stage 2 hypertension at screening (*p* < 0.001) than those who did not link to NCD care. At their first clinic visit, 294/2038 (15%) of individuals were on blood pressure lowering medication, 714/2038 (35%) had Stage 2 hypertension, and 123/2038 (6%) were also being treated for diabetes. Among the 89 HIV-infected participants who linked to NCD care, 31 (36.9%) had HIV RNA < 400 copies/mL at baseline [Table 1].

### Hypertension care

Overall, the 2038 patients who linked to HTN care made 16,253 total visits over 3 years of follow-up; of these, 1587 patients contributed 15,653 visits after the initial linkage visit [Table 2]. The median duration of between follow-up visits was 583 days (IQR 84–1122 days) among HIV-infected patients and 742 days (IQR 48–1198 days) among the HIV uninfected participants. Actual visits occurred > 14 days after the scheduled date (late visits) at 18% (2975/2038) of visits. Among visits where hypertension was uncontrolled, 65% (6229/8179) of subsequent follow-up visits were scheduled at > 30 days (less frequently than guidelines). Among visits where hypertension was controlled, 43% (3197/7474) of subsequent follow-up were scheduled at < 84 days (more frequently than clinical guidelines) due to drug stock outs. Patients’ blood pressure was controlled at 46% (44–48%) of follow-up visits.

**Table 2:** Follow-up clinic visits among those who linked to hypertension care after population level screening (*N* = 2038)

	HIV+ (*n* = 89)	HIV-(*n* = 1949)	Overall (*N* = 2038)
Total number of follow-up visits	580	15,073	15,653
Median duration of follow-up (days) (IQR)	583 (84, 1122)	742 (48, 1198)	738 (50, 1195)
Interval in days between scheduled visits [Median (IQR)]	59 (29, 84)	56 (29, 84)	56 (29, 84)
Interval in days between actual visits [Median (IQR)]	65 (42, 90)	63 (35, 91)	63 (35, 91)
Interval between scheduled visits			
More frequent than clinical indication	141 (24%)	3056 (20%)	3197 (20%)
Less frequent than clinical indication	216 (37%)	6013 (39%)	6229 (39%)
Late visits^a^ [%, median (IQR)]	150 (24%)	2825 (18%)	2975 (18%)
Follow-up visits with controlled blood pressure	301 (48%)	7173 (46%)	7474 (46%)
Follow-up visits with controlled blood pressure on 2 consecutive visits	182 (34%)	4160 (29%)	4342 (29%)
Visits with no blood pressure measurement	33 (3.5%)	609 (3.5%)	641 (3.5%)

^a^late visit defined as > 14 days after scheduled visit

Approximately two thirds of the patients (1333/2038) were treated with blood pressure lowering medicines and of these 65% (909/1333) were treated with 2 or more medications. Almost all (1290/1333, 96%) of those treated with medications were prescribed bendroflumethiazide (a thiazide diuretic) and 902/1333 (68%) were treated with nifedipine (a dihydropyridine calcium channel blocker). The remaining third who were never prescribed blood pressure lowering medication were managed though counseling on lifestyles changes, including decreasing salt intake, exercise, and alcohol consumption [Table 3].

**Table 3:** Hypertension treatment among those receiving care in clinic (N = 2038)

	HIV+ (*n* = 89)	HIV-(*n* = 1949)	Overall (*N* = 2038)
*Treatment*			
Blood pressure lowering medications [n(%)]	60 (67%)	1275 (65%)	1333 (65%)
Lifestyle changes [n(%)]	29 (33%)	674 (35%)	703 (35%)
Number of Blood Pressure Medications			
1	21 (23%)	404 (21%)	424 (21%)
2	23 (25%)	723 (37%)	745 (37%)
> = 3	6 (7%)	158 (8%)	164 (8%)
Frequency of prescribed medications [n(%)]			
Bendroflumethiazide	46 (54%)	1245 (98%)	1290 (96%)
Nifedipine	29 (33%)	875 (67%)	902 (68%)
Atenolol	10 (13%)	246 (19%)	256 (19%)
Captopril	8 (9%)	216 (17%)	224 (17%)
Furosemide	1 (2%)	38 (3%)	39 (3%)
Propranolol	0	26 (2%)	26 (2%)
Amlodipine	1 (2%)	24 (2%)	25 (2%)

### Predictors of hypertension control

After adjusting for age, sex, comorbid diabetes, hypertension stage at previous visit, medication prescribed at previous visit, HIV status, and clinic, scheduled visit interval outside of clinical indication was significantly associated with blood pressure control. Scheduled visit interval more frequent than clinical indication for patients with controlled HTN was associated with lower HTN control (aOR = 0.89; 95% CI 0.79–0.99). Men (aOR 0.88; 95% CI 0.78–0.99) and patients age 50 years and older (aOR 0.83; 95% CI 0.73–0.95) were less likely to have controlled blood pressure at follow-up visits than women and patients less than 50 years old. Time in care (in months) was associated with higher odds of blood pressure control (aOR 1.03; 95% CI 1.03–1.04). HIV-infected patients were more likely than uninfected patients to have controlled blood pressure at follow-up visits (aOR 1.28; 95% CI 1.00–1.77) [Table 4]. When 2 consecutive clinic visits with controlled hypertension was the outcome age > = 50 years (aOR 0.73; 95% CI 0.58–0.93) and scheduled visit interval more frequent than clinical indication for patients with controlled HTN (aOR 0.80; 95% CI 0.52–0.99) were associated with lower odds of HTN control [Table 5].

**Table 4:** Predictors of hypertension control among patients who linked to hypertension care following population-level screening and had at least one subsequent follow-up visit (*n* = 1587)

	Unadjusted OR (95% CI) for HTN control	*p*-value	Adjusted OR (95% CI) for HTN control	*p* value
Sex		0.08		0.03
Female	1.0		1.0	
Male	0.87 (0.75, 1.01)		0.88 (0.78, 0.99)	
Age		0.02		0.01
< 50 years	1.0		1.0	
> = 50 years	0.82 (0.69, 0.97)		0.83 (0.73, 0.95)	
Diabetes present		0.9		0.8
No	1.0		1.0	
Yes	1.02 (0.80, 1.31)		0.98 (0.80, 1.19)	
Time since first clinic visit (months)	1.03 (1.03, 1.03)	< 0.001	1.02 (1.01, 1.02)	< 0.001
Hypertension Stage at previous visit				
0	1.0	< 0.001	1.0	< 0.001
1	0.51 (0.46, 0.57)		0.63 (0.55, 0.71)	
2	0.26 (0.23, 0.29)		0.33 (0.28, 0.38)	
Anti-hypertensive Medication prescribed at previous visit		0.02		0.15
Yes	1.26 (1.07, 1.49)		1.09 (0.94, 1.26)	
No	1.0		1.0	
HIV Status		0.04		0.11
Known-infected	1.35 (1.02, 1.78)		1.28 (0.95, 1.71)	
Known-uninfected	1.0		1.0	
Scheduled visit interval		0.03		0.05
Per HTN treatment guidelines*	1.0		1.0	
More frequently than guidelines*	0.81 (0.75, 0.87)		0.89 (0.79, 0.99)	
Clinic		< 0.001		< 0.001
Bugamba	0.65 (0.50, 0.85)		0.67 (0.54, 0.85)	
Kameke	4.37 (3.19, 6.00)		3.79 (2.86, 5.04)	
Kamuge	1.39 (1.10, 1.75)		1.64 (1.34, 2.00)	
Kazo	1.64 (1.18, 2.29)		1.60 (1.20, 2.11)	
Merikit	1.81 (1.37, 2.31)		1.74 (1.38, 2.19)	
Mitooma	1.0		1.0	
Muyembe	1.91 (1.45, 2.52)		1.89 (1.50, 2.37)	
Nankoma	1.91 (1.39, 2.64)		1.89 (1.50, 2.37)	
Rubaare	1.07 (0.78, 1.48)		1.10 (0.85, 1.44)	
Ruhoko	1.26 (0.91, 1.73)		1.16 (0.91, 1.49)	

*Guidelines are to schedule follow-up visit 4 weeks later for patients with uncontrolled hypertension (stage 1 or stage 2) and every 3 months for patients with controlled hypertension (stage 0) at previous visit. More frequently than guidelines is a scheduled follow-up visit < 84 days when blood pressure is controlled (SBP < 140 and DBP < 90)

**Table 5:** Predictors of hypertension control at two consecutive visits among patients who linked to hypertension care following population-level screening and had at least two subsequent follow-up visits (*n* = 1327)

	Unadjusted OR (95% CI) for HTN control	*p*-value	Adjusted OR (95% CI) for HTN control	*p* value
Sex		0.08		0.09
Female	1.0		1.0	
Male	0.82 (0.65, 1.02)		0.83 (0.68, 1.03)	
Age		0.008		0.009
< 50 years	1.0		1.0	
> =50 years	0.72 (0.56, 0.92)		0.73 (0.58, 0.91)	
Diabetes present		0.99		0.99
No	1.0		1.0	
Yes	0.99 (0.71, 1.39)		1.01 (0.72, 1.39)	
Time since first clinic visit (months)	1.03 (1.03, 1.04)	< 0.001	1.03 (1.02, 1.04)	< 0.001
Hypertension Stage at previous visit		< 0.001		< 0.001
0	1.0		1.0	
1	0.87 (0.69, 1.08)		0.89 (0.78, 0.99)	
2	0.42 (0.07, 2.52		0.79 (0.67, 0.91)	
Anti-hypertensive Medication prescribed at previous visit		0.009		0.28
Yes	0.74 (0.59, 0.93)	0.03	1.03 (0.89, 1.14)	0.21
No	1.0		1.0	
HIV Status		0.06		0.07
Known-infected	1.81 (0.97, 3.41)		1.54 (0.93, 2.65)	
Known-uninfected	1.0		1.0	
Scheduled visit interval				
Per HTN treatment guidelines	1.0	< 0.001	1.0	0.02
More frequently than guidelines	0.68 (0.37, 0.98)		0.80 (0.52, 0.99)	
Clinic				
Bugamba	0.36 (0.24, 0.53)		0.46 (0.31, 0.68)	
Kameke	6.98 (4.62, 10.54)		5.05 (3.30, 7.72)	
Kamuge	1.34 (0.97, 1.85)		1.21 (0.88, 1.68)	
Kazo	0.74 (0.45, 1.22)		0.77 (0.46, 1.31)	
Merikit	1.77 (1.19, 2.61)		1.10 (0.74, 1.61)	
Mitooma	1.0		1.0	
Muyembe	2.32 (1.60, 3.37)		1.24 (0.85, 1.81)	
Nankoma	2.76 (1.80, 4.22)		2.30 (1.48, 3.56)	
Rubaare	0.87 (0.56, 1.35)		0.80 (0.50, 1.28)	
Ruhoko	1.15 (0.74, 1.78)		0.45 (0.29, 0.69)	

## Discussion

To work towards hypertension control in SSA we need to optimize health systems for chronic care delivery. In this study of hypertension outcomes among patients referred to integrated chronic disease care after population-wide screening we found that blood pressure control increased more than threefold from 15% at baseline. Nevertheless, blood pressure was controlled in slightly less than half (46%) of all follow-up visits. We identified a modifiable systems factor, more frequent clinic visits precipitated by drug stock outs, as one of the barriers to hypertension control.

Achieving hypertension control at a population level starts with screening and linkage to clinical care. After population-based screening, 45% of patients with prevalent hypertension were linked to NCD care. This number may underestimate true linkage, however, as individuals who were normotensive on presentation to clinic and not enrolled in NCD care were not counted, however they still fall short of ideal. Linkage to NCD care following screening remains a challenge across the region – a recent meta-analysis on hypertension in SSA estimated only 14–22% patients were in care following hypertension diagnosis [5]. Our population-based, multi-disease approach may have increased linkage by increasing health-seeking behavior in the communities; however, additional efforts targeted towards engagement in NCD care are needed.

Our population who sought care were approximately two thirds female, while population-based screening demonstrated higher prevalence among men [26]. Our clinic population that is enriched for women is similar to other reported clinic populations [7] likely reflects the health care seeking behavior of women in a health care system created primarily for women and children. Only 15% were on blood pressure lowering medications at baseline, reflecting the large burden of undiagnosed disease in this part of the world. Over 1/3 had stage 2 hypertension at the time of screening suggesting this undiagnosed burden is severe.

We found that hypertension control was achieved at 46% of follow-up visits. This is similar to findings of a multisite population population-based screening across 6 sites in South Africa, Kenya, Ghana, Burkina Faso, Sierra Leone where HTN control in 47% of those in treatment [8] and slightly higher than 37% hypertension control among those on blood pressure lowering medication in a population based study in Malawi [6]. However, we measure hypertension control over time rather than a single point estimate, which provides a more complete picture of hypertension control in the clinic community.

Similar to findings that improved HIV outcomes are associated with extended intervals between scheduled clinic visits [17, 18], we found that patients whose scheduled visit intervals were more frequent than clinical guidelines had worse BP control. Medication stock levels contributed to more frequent scheduled visits (i.e. < 12 weeks despite hypertension control) at 20% of visits. This finding provides critical information for targeting reduced patient visits per clinical guidelines for efficient chronic care for stable controlled patients and ensuring the clinic infrastructure, including drug supply, can support less frequent visits. More reliable drug supply chains for NCDs will be crucial to this effort [27].

Patients with dual diagnoses of HIV and hypertension were more likely to achieve normal blood pressure over time than those patients receiving care for hypertension only. Hypertension care was integrated into HIV clinic visits preventing redundant visits and HIV-infected patients also received extensive counseling about daily medication adherence and retention support which may have led to increased adherence among HIV-infected patients. These tools can be adapted to support engagement in care for other chronic disease to improve outcomes.

Other integration initiatives can inform the successful integration of HIV and hypertension care. Integrated TB and HIV care leads to decreased both HIV and TB-associated morbidity and mortality [28]. Co-location of services is associated with fewer delays in starting ART and greater uptake of ART among HIV/TB co-infected patients [29–31] versus referral to a separate facility for TB or HIV care. Similarly, integration of family planning and HIV counseling and testing increases uptake of both among post-partum women when compared to stand-alone service delivery [32]. We provided integrated HIV and hypertension care under the same roof enabling “one-stop” shopping for patients. As hypertension and chronic care for other NCDs is integrated with HIV chronic care across SSA, co-located services, a well-trained workforce, and clinic infrastructure will likely be crucial to successful treatment of both.

Our data only captures individuals who are in care for hypertension treatment; outcomes following transfer or loss to care were not assessed. Nonetheless, understanding blood pressure control among those individuals receiving treatment is important to improving outcomes within the health care system. More detailed analysis of the effect of class of antihypertensive treatment would provide additional insight into mechanisms of blood pressure control, however the focus of this current analysis is on individual clinical factors and health systems factors that contribute to hypertension control. Additionally, blood pressure measured at clinic represents a single point in time and we thus have an incomplete picture of overall hypertension control in this population. Clinic-based blood pressure measurements potentially misclassify individuals with white coat hypertension (where an individual presents as hypertensive in clinic but is normotensive out of clinic) or masked hypertension (where an individual presents as normotensive in clinic but has elevated blood pressure on ambulatory or home BP monitoring) and the prevalence in SSA is estimated to be 15 and 11% respectively [33]. However, these misclassifications would likely bias our results towards the null. Finally, adherence to blood pressure lowering medications was not evaluated in this analysis, however adherence to medication is likely reflected in the blood pressure measurements performed in clinic. Future work will probe adherence and challenges to adherence among those with poor hypertension control.

## Conclusion

Our study registered successes in population level screening for HIV and hypertension, in linkage to integrated chronic disease care and hypertension control for both HIV positive and HIV negative patients with hypertension. Our study contributes evidence to realize effective responses for HIV care and emerging NCDs, including hypertension, in SSA. However, there is need to continue to optimize the integrated care model to achieve ideal patient outcomes.

## Additional files

Additional file 1: Hypertension Management Algorithm (PDF 70 kb)

Additional file 2: Hypertension Drug Use Algorithm (PDF 172 kb)

## Abbreviations

ART: Antiretroviral therapy
BP: Blood Pressure
CHC: Community health campaign
DBP: Diastolic blood pressure
HIV: Human immunodeficiency virus
HTN: Hypertension
NCD: Non-communicable disease
PLHIV: People living with HIV
SBP: Systolic blood pressure
SSA: Sub-Saharan Africa
TB: Tuberculosis
WHO: World Health Organization
