# Supplementary material for: Hypertension control in integrated HIV and chronic disease clinics in Uganda in the SEARCH study
Source: BMC Public Health. 2019 May 6;19:511. doi: 10.1186/s12889-019-6838-6 (PMC6501396; doi:10.1186/s12889-019-6838-6)
Supplement: Supplementary file 1 — Hypertension Management Algorithm (PDF 70 kb) [file 12889_2019_6838_MOESM1_ESM.pdf]

Appendix 1

Hypertension Management Algorithm

Measure **BP** in **ALL** adults

SBP >140 or DBP >90

Check again 3 times at 1 minute intervals

If any screening BP has SBP<140 and DBP<90 → Evaluate again after 6 month

High BP after 3 screenings

Ask about symptoms

Shortness of breath on small exercises, feet swollen, chest Pain, limb weakness/paralysis, blurred vision, headache or convulsions.

If "Yes" to any symptoms → Call ART Coordinator or Refer patient to hospital

No symptoms

Check cardiovascular risk factors

a) Proteinuria (on dipstick)

b) Diabetic (RBS>11.1mmoles)

If "Positive" on either test → Call ART Coordinator or Refer patient to hospital

No risk factors

SBP 140-159 and/or DBP 90-99	SBP 160-179 and/or DBP 100-109	SBP >180 or DBP > 110
3-month trial of **lifestyle changes**:<br><br>Weight reduction, increase physical exercise, decrease dietary salt intake, decrease alcohol consumption, stop smoking promote fresh fruits and vegetables intake, fatty food limitation<br><br>**Follow up in 3 months** - If BP remains high then start medications	**lifestyle changes**:<br>Weight reduction, increase physical exercise, decrease dietary salt intake, decrease alcohol consumption, stop smoking, promote fresh fruits and vegetables intake, fatty food limitation<br><br>**PLUS**<br><br>**Medications**<br>(Follow treatment algorithm)	Call ART Coordinator<br>or<br>Refer patient to hospital

June 24, 2015
