# Supplementary material for: Hypertension control in integrated HIV and chronic disease clinics in Uganda in the SEARCH study
Source: BMC Public Health. 2019 May 6;19:511. doi: 10.1186/s12889-019-6838-6 (PMC6501396; doi:10.1186/s12889-019-6838-6)
Supplement: Supplementary file 2 — Hypertension Drug Use Algorithm (PDF 172 kb) [file 12889_2019_6838_MOESM2_ESM.pdf]

Appendix 2

Hypertension Drug Use Algorithm

Initial medication regimen

Bendroflumethiazide 5mg daily

Follow-up in **4 weeks**

↓

Target BP not achieved?

Increase
Bendroflumethiazide 10mg
Follow-up in **4 weeks**

↓

Target BP not achieved?

Bendroflumethiazide 10 mg + Nifedipine 20 mg BID

Follow-up in **4 weeks**

↓

Target BP not achieved?

Bendroflumethiazide 10 mg + Nifedipine 20 mg BID +
Add **Captopril 25 mg BID**

Follow-up in **4 weeks**

↓

Call ART Coordinator
or
Refer patient to the hospital

BP GOALS

Age <60 or TOD: SBP <140 and DBP <90

Age ≥60: SBP <150 and DBP<90

TARGET BP ACHIEVED?

Prescribe same treatment and follow up every 3 months

Measure BP & weight at each visit

Medication Adherence:
Always check for drug adherence **at each visit.** Ask whether patient has **taken medication on each of the last three days**. Provide **counseling** if patient has **missed any dose** in the last three days.

Lifestyle and Behavior
Review life style and Behavior. Provide Counseling if still present

Medication Tolerability:
If patient is having **side effects**, change to another medication. Alternatively, call ART coordinator or refer patient to hospital

Key
BID-Twice a day; SB -Systolic; DBP-Diastolic BP; TOD-target organ damage (Heart failure, kidney failure, stroke, myocardial infarction etc)

June 23, 2015
